# The relationship between prognosis and temporal muscle thickness in 102 patients with glioblastoma

**DOI:** 10.1038/s41598-024-64947-z

**Published:** 2024-06-17

**Authors:** Jinhai Tang, Zhenghao Dong, Lei Yang, Ping Yang, Wanying Zhao, Lvdan Deng, Juan Xue, Yijie Cui, Qizheng Li, Lufan Tang, Junxiu Sheng, Yu Zhang, Huimin Zhang, Tongtong Chen, Bin Dong, Xiupeng Lv

**Affiliations:** 1 Department of Radiation Oncology, The First Affiliated Hospital of Dalian Medical University, Dalian, Liaoning China; 2 Department of Thoracic Surgery, West China Hospital of Sichuan University, Chengdu, Sichuan China; 3 Department of Radiation Oncology, The Fourth Affiliated Hospital of Hebei Medical University, Shijiazhuang, Hebei China; 4 Department of Neurosurgery, The First Affiliated Hospital of Dalian Medical University, Dalian, Liaoning China

**Keywords:** Cancer, Neuroscience, Biomarkers, Medical research, Neurology, Oncology, Signs and symptoms

## Abstract

Temporal muscle thickness measured on 3D MRI has recently been linked to prognosis in glioblastoma patients and may serve as an independent prognostic indicator. This single-center study looked at temporal muscle thickness and prognosis in patients with primary glioblastoma. Overall survival was the major study outcome. For a retrospective analysis from 2010 to 2020, clinical data from 102 patients with glioblastoma at the Department of Oncology Radiotherapy of the First Affiliated Hospital of Dalian Medical University were gathered. Fifty-five cases from 2016 to 2020 contained glioblastoma molecular typing data, of which 45 were IDH wild-type glioblastomas and were analysed separately. TMT was measured on enhanced T1-weighted magnetic resonance images in patients with newly diagnosed glioblastoma.Overall patient survival (OS) was calculated by the Kaplan–Meier method and survival curves were plotted using the log-rank-sum test to determine differences between groups, and multifactorial analyses were performed using a Cox proportional-risk model.The median TMT for 102 patients was 6.775 mm (range: 4.95–10.45 mm). Patients were grouped according to median TMT, and the median overall survival (23.0 months) was significantly longer in the TMT > median group than in the TMT median group (P 0.001; Log-rank test). Analysing 45 patients with IDH wild type alone, the median overall survival (12 months) of patients in the TMT > median group was significantly longer than that of patients in the TMT ≤ median group (8 months) (P < 0.001; Log-rank test).TMT can serve as an independent prognostic factor for glioblastoma.

## Introduction

Glioblastomas (GBM), which are invasive original cerebral malignant tumors with a high morbidity and mortality rate, account for approximately 50% of all gliomas^1^. The Stupp regimen, which comprises maximum surgical resection followed by radiation therapy along with temozolomide (TMZ) chemotherapy, is presently regarded the preferred treatment for GBM^2^. Despite successful intervention and treatment, the prognosis for patients is unfavorable, with a median survival rate of less than 2 year^3^.

Some of the characteristics that have been shown to affect the prognosis of GBM patients are age, preoperative performance status, tumor location, degree of resection, adjuvant therapy, and genetic features^4,5^. Recent studies on prognostic factors in patients with glioma have taken into account more other variables, including the ratio of albumin to globulin, the ratio of neutrophils to lymphocytes, the prognostic trophic index, and the ratio of platelets to lymphocytes^6^. Preoperative functional status of the patient is an essential reference that impacts prognosis and can be examined clinically before to surgery. However, the doctor's subjective judgment influences the clinical assessment of the client, resulting in high interobserver variability and a lack of objectivity in the clinical assessment^7^. As a result, there is an urgent need for objectively measured measures to estimate patients' frailty in order to improve prognosis assessment. Skeletal muscle mass can be used as an objective measure to determine a patient's physical state.

Sarcopenia refers to the loss of skeletal muscle mass and is a critical component of cancer-related cachexia as well as an important prognostic factor in surgical oncology^8,9^. Temporal muscle thickness (TMT) has been shown to be a suitable alternative to evaluating skeletal muscle mass in determining sarcopenia^10,11^. According to many studies that have looked at the association between survival time and TMT in patients with brain metastases in recent years, TMT may be used as an independent criterion for predicting frailty and survival in patients with brain malignancies^12,13^.

Recently the relationship between temporal muscle thickness and the prognosis of glioblastoma patients has become a research hotspot, and some scholars have pointed out that temporal muscle thickness can be used as an independent risk factor for the prognosis of glioblastoma patients, which can help the assessment of the survival of the patients^14,15^, but the conclusion of this is still controversial. Therefore, the main purpose of this study was to investigate the prognostic role of temporal muscle thickness in glioblastoma patients, whether it can be used as an independent predictor for glioblastoma patients, and furthermore, to investigate the prognostic relationship between TMT and IDH wild-type glioblastoma patients in the era of molecular typing.

## Results

### Patient demographics and clinical characteristics

A total of 102 patients were included in the study. The demographic clinical characteristics of the patients are shown in Table 1. Among them, 56 (54.9%) were male and 46 (45.1%) were female, the median age was 59.5 years (range: 31–77 years), the median survival time was 14 months (range: 2–85 months), the supratentorial tumors were 95 (93.1%) and the infratentorial tumors were 7 (6.9%), the maximum diameter of the tumors was < 5 cm in 52 cases (51%), the maximum diameter of the tumors was ≥ 5 cm in 50 cases (49%) The median KPS score was 70 (range: 50–90), the number of patients who were operated on and received postoperative temozolomide-synchronized radiotherapy was 80 (78.4%), and the median TMT of 102 patients was 6.775 mm (range: 4.950–10.45 mm), the median for male patients was 6.825 mm (range: 5.20–10.45 mm), and the median for female patients was 6.725 mm (range: 4.95–9.70 mm). (Table 1).

**Table 1:** Baseline data on 102 patients with glioblastoma.

Characteristic	Description	Value
Median age at the diagnosis of GBM	Median, years (range)	59.5(31–77)
Sex		
Males	N (%)	56 (54.9%)
Females	N (%)	46 (45.1%)
Tumor location		
Supratentorial	N (%)	95 (93.1%)
Infratentorial	N (%)	7 (6.9%)
Tumor size		
< 5 cm	N (%)	52 (51%)
≥ 5 cm	N (%)	50 (49%)
KPS	Median (range)	70 (50–90)
Adjuvant radiotherapy with concomitant temozolomide		
With	N (%)	80 (78.4%)
Without	N (%)	22 (21.6%)
TMT	Median, millimeter (range)	6.775 (4.950–10.45)
Overall survival (in months)	Median, months (range)	14 (2–85)

Of the 55 patients with molecular typing information, 45 of them were patients with IDH wild-type glioblastoma. Their demographic and clinical characteristics are shown in Table 2. 18 (40.0%) were male and 27 (60.0%) were female, the median age was 63 years (range: 35–77 years), the median survival time was 10 months (range: 4–30 months), supratentorial tumours were 43 (95.6%) and infratentorial tumours were 2 (4.4%), the maximal diameter of the tumour was < 5 cm in 29 cases (64.4%), tumour maximum diameter ≥ 5 cm in 16 cases (35.6%), median KPS score was 70 (range: 50–80), the number of patients who were operated and underwent postoperative temozolomide synchronous radiotherapy was 40 (88.9%), the number of patients who were completely resected was 16 (35.6%), and the number of patients who were incompletely resected was 29 (64.4%), and the number of patients who were methylated for MGMT was 6 (13.3%), MGMT unmethylated in 39 cases (86.7%), TP53 mutated in 13 cases (28.9%), TP53 unmutated in 32 cases (71.1%), and the median TMT of 45 patients was 6.60 mm (range: 5.15–9.70 mm), (Table 2).

**Table 2:** Baseline data on 45 patients with glioblastoma of the IDH wild type.

Characteristic	Description	Value
Median age at the diagnosis of GBM	Median, years (range)	63(35–77)
Sex		
Males	N (%)	18 (40.0%)
Females	N (%)	27 (60.0%)
Tumor location		
Supratentorial	N (%)	43 (95.6%)
Infratentorial	N (%)	2 (4.4%)
Tumor size		
< 5 cm	N (%)	29 (64.4%)
≥ 5 cm	N (%)	16 (35.6%)
Extent of resection		
Gross total resection	N (%)	16 (35.6%)
Subtotal resection	N (%)	29 (64.4%)
KPS	Median (range)	70 (50–80)
Adjuvant radiotherapy with concomitant temozolomide		
With	N (%)	40 (88.9%)
Without	N (%)	5 (11.1%)
TMT	Median,millimeter (range)	6.60 (5.15–9.70)
MGMT		
Methylated	N (%)	6 (13.3%)
Unmethylated	N (%)	39 (86.7%)
TP53		
Mutant	N (%)	13 (28.9%)
Non-mutant	N (%)	32 (71.1%)
Overall survival (in months)	Median,months (range)	10 (4–30)

### Correlation of TMT with survival rate

**Figure 1:**
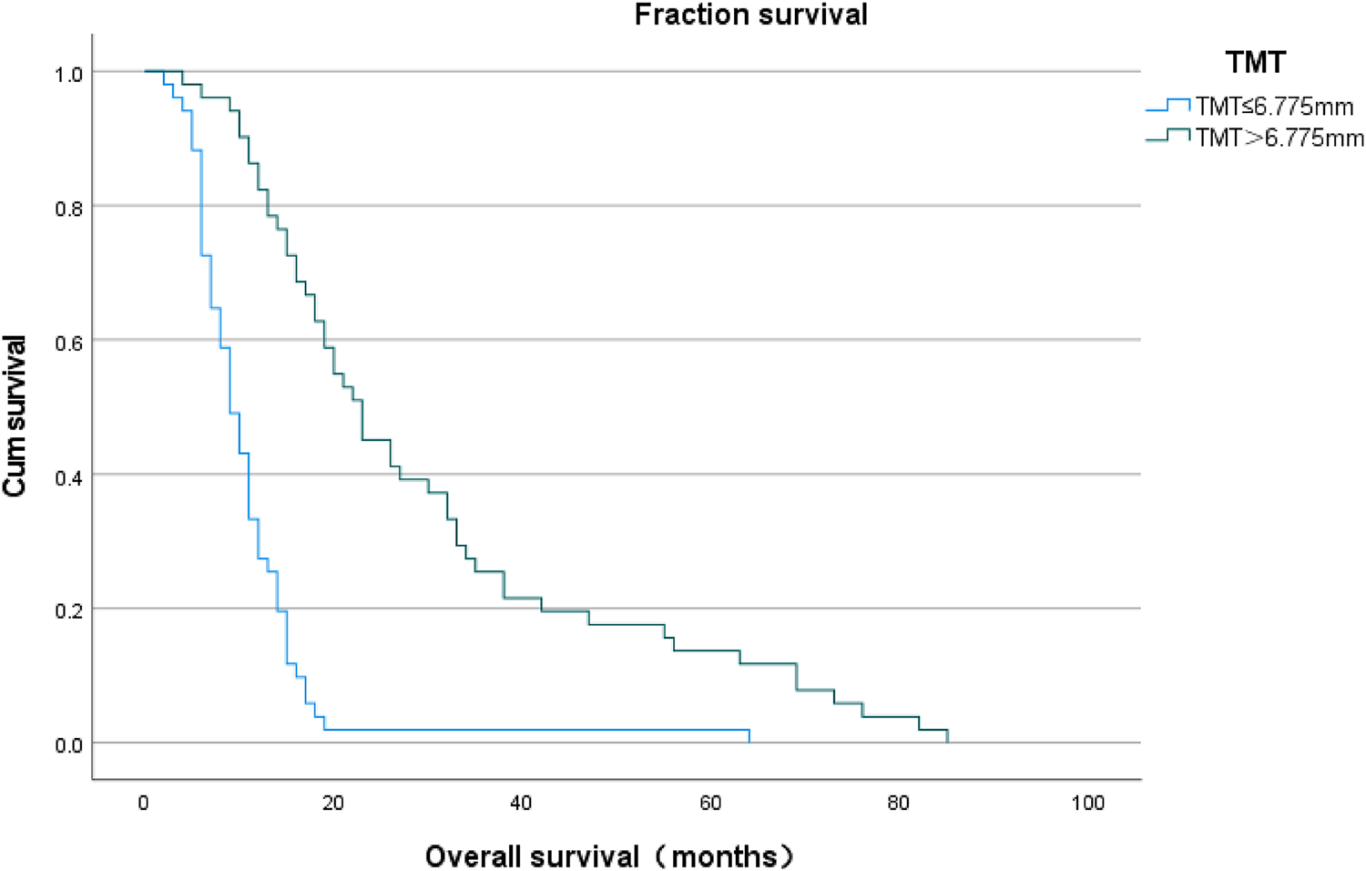
Kaplan–Meier analysis of overall survival according to median temporal muscle thickness (TMT) in 102 patients with glioblastoma(GBM). Median survival for TMT ≤ 6.775 was 9.0 months and 23.0 months for TMT > 6.775 (P < (P 5 h).

The median TMT of 102 patients was 6.775 mm (range: 4.95–10.45 mm) and this median value was used as the cutoff value for this study, with a median of 6.825 mm (range: 5.20–10.45 mm) for male patients and 6.725 mm (range: 4.95–9.70 mm) for female patients. Grouped by median TMT, 51 of them were assigned to the thinner group and the other 51 to the thicker group, and the clinical characteristics of the patients in these groups are indicated in Table 3. The median overall survival of the patients in the TMT > median group (23.0 months) was significantly increased (P < 0.001; Log-rank test) compared with that of the patients in the ≤ median group (9 months), as indicated in Fig. 1.

**Table 3:** Correlation of TMT with Clinicopathological Data in 102 patients.

Characteristic		TMT > 6.775 mm	TMT ≤ 6.775 mm
No. of patients		51	51
Average age(years) ± SD		54.18 ± 13.18	60.98 ± 10.67
Sex	Males	29	27
	Females	22	24
Tumor location	Supratentorial	48	47
	Infratentorial	3	4
Tumor size	< 5 cm	29	23
	≥ 5 cm	22	27
Median KPS (range)		80 (50–90)	70 (50–80)
Adjuvant radiotherapy with concomitant temozolomide	With	47	33
	Without	4	18
Average overall survival (in months) ± SD		30.02 ± 21.23	10.90 ± 8.64

**Figure 2:**
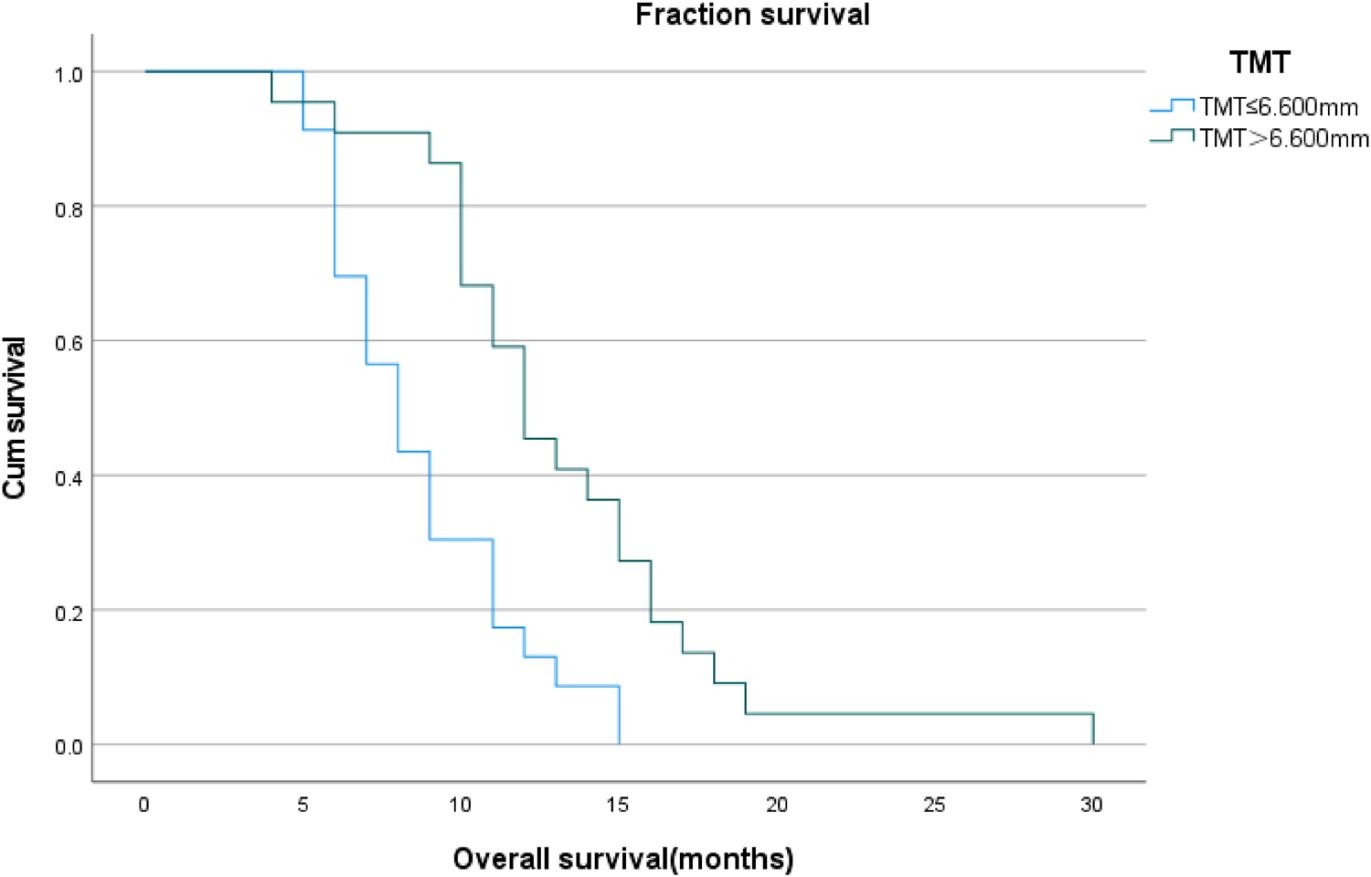
Kaplan–Meier analysis of overall survival according to median temporal muscle thickness (TMT) in 45 patients with IDH wild-type glioblastoma(GBM). Median survival for TMT ≤ 6.600 was 8.0 months and 12.0 months for TMT > 6.600 (P < 0.001).

Forty-five patients with IDH wild-type glioblastoma were analysed individually. The median TMT of the 45 patients was 6.600 mm (range: 5.150–9.700 mm).The median TMT of male patients was 6.575 mm (range: 5.30–8.50 mm) and that of female patients was 6.60 mm (range: 5.15–9.70 mm).The patients were grouped according to the median TMT grouped the patients, 22 of them were assigned to the thinner group and the other 23 to the thicker group, and the clinical characteristics of the patients in these groups are represented in Table 4. Survival analysis by the Kaplan–Meier method showed a significant increase in the median overall survival of the patients in the TMT > median group (12 months) compared to the median overall survival of the patients in the TMT ≤ median group (8 months), and according to the log-rank sum test, there was a statistical difference between the two groups (P < 0.001; Log-rank test), as indicated in Fig. 2.

**Table 4:** Correlation of TMT with Clinicopathological Data in 45 patients.

Characteristic		TMT > 6.60 mm	TMT ≤ 6.60 mm
No. of patients		22	23
Average age(years) ± SD		59.77 ± 8.89	62.04 ± 11.20
Sex	Males	9	9
	Females	13	14
Tumor location	Supratentorial	21	22
	Infratentorial	1	1
Tumor size	< 5 cm	16	13
	≥ 5 cm	6	10
Median KPS (range)		70 (50–80)	70 (50–80)
Adjuvant radiotherapy with concomitant temozolomide	With	20	20
	Without	2	3
Average overall survival (in months) ± SD		13.18 ± 5.28	8.70 ± 3.00
Extent of resection	Gross total resection	11	5
	Subtotal resection	11	18
MGMT	Methylated	5	1
	Unmethylated	17	22
TP53	Mutant	6	7
	Non-mutant	16	16

### Multivariate analysis of factors affecting survival in glioblastoma

In 102 patients, age at diagnosis, gender, tumour site, maximum tumour diameter, extent of tumour resection, KPS score, postoperative radiotherapy with simultaneous temozolomide chemotherapy, and TMT were subjected to univariate analysis. Including TMT, radiotherapy and simultaneous temozolomide chemotherapy, age at diagnosis, and KPS score in the multifactorial analysis, multivariate survival analysis using Cox regression model showed (Table 5) that TMT (HR 0.547; 95% CI 0.389 to 0.769; P < 0.001), radiotherapy and simultaneous temozolomide chemotherapy (HR 0.477; 95% CI 0.272 to 0.834; P = 0.009) were significantly associated with survival and were independent risk factors affecting the prognosis of GBM patients. Age at diagnosis (HR 1.013; 95% CI 0.996 to 1.031; P = 0.142) and KPS score (HR 0.981; 95% CI 0.960 to 1.002; P = 0.075), on the other hand, had no effect on survival of GBM patients (Table 6).

**Table 5:** Univariate Analysis and Multivariate Cox regression analysis for progression free survival in 102 patients.

Characteristics	Univariate analysis		Multivariate analysis	
	HR (95% *CI*)	*P*-value	HR (95% *CI*)	*P*-value
Age (> 59.5 years vs. ≤ 59.5 years)	1.029 [1.013, 1.046]	< 0.001	1.013 [0.996, 1.031]	0.142
Sex (female vs.male)	0.956 [0.642, 1.422]	0.823		
Tumor location (supratentorial vs.Infratentorial)	1.069 [0.492, 2.322]	0.866		
Tumor size (≥ 5 cm vs. < 5 cm)	1.153 [0.780, 1.705]	0.474		
Type of surgery (Gross total resection vs.Subtotal resection)	0.839 [0.553, 1.272]	0.408		
KPS (≥ 70 vs. < 70)	0.953 [0.936, 0.971]	< 0.001	0.981 [0.960, 1.002]	0.075
Received concomitant radiochemotherapy and adjuvant chemotherapy(Yes vs.No)	0.271 [0.160, 0.457]	< 0.001	0.477 [0.272, 0.834]	0.009
TMT > 6.775 mm vs. ≤ 6.775 mm)	0.395 [0.302, 0.517]	< 0.001	0.547 [0.389, 0.769]	< 0.001

**Table 6:** Univariate Analysis and Multivariate Cox regression analysis for progression free survival in 45 patients.

Characteristics	Univariate analysis		Multivariate analysis	
	HR(95%*CI*)	*P-value*	HR(95%*CI*)	*P-value*
Age (> 59.5 years vs. ≤ 59.5 years)	1.021 [0.991, 1.053]	0.170		
Sex(female vs.male)	1.077 [0.589, 1.969]	0.809		
Tumor location(supratentorial vs.Infratentorial)	1.835 [0.439, 7.662]	0.405		
Tumor size(≥ 5 cm vs. < 5 cm)	1.848 [0.974, 3.508]	0.060	1.608 [0.770, 3.360]	0.206
Type of surgery (Subtotal resection vs.Gross total resection)	3.279 [1.617,6.650]	< 0.001	3.517 [1.459, 8.476]	0.005
KPS (≥ 70 vs. < 70)	0.966 [0.935, 0.997]	< 0.032	0.955 [0.919, 0.992]	0.019
Received concomitant radiochemotherapy and adjuvant chemotherapy(Yes vs.No)	0.069 [0.018, 0.257]	< 0.001	0.110 [0.029, 0.422]	0.001
TMT (> 6.60 mm vs. ≤ 6.60 mm)	0.325 [0.195, 0.543]	< 0.001	0.439 [0.257, 0.749]	0.003
MGMT (Unmethylated vs.Methylated)	3.282 [1.263, 8.534]	0.015	2.597 [0.799,8.441]	0.112
TP53 (Mutant vs.Non-mutant)	1.930 [0.976, 3.816]	0.059		

In 45 patients with IDH wild-type glioblastoma, age at diagnosis, gender, tumour site, maximum tumour diameter, degree of tumour resection, KPS score, postoperative radiotherapy with simultaneous temozolomide chemotherapy, TMT, MGMT, and TP53 were included in the univariate analysis. TMT, radiotherapy with simultaneous temozolomide chemotherapy, age at diagnosis, KPS score, degree of tumour resection, maximum tumour diameter, and MGMT were included in the multifactorial analysis, and multivariate survival analysis using Cox regression models showed that TMT (HR 0.439; 95% CI 0.257 to 0.749; P = 0.003), radiotherapy with simultaneous temozolomide chemotherapy (HR 0.110; 95% CI 0.029 to 0.422; P = 0.001), KPS score (HR 0.955; 95% CI 0.919 to 0.992; 0.019), and the extent of tumour resection (HR 3.517; 95% CI 1.459 to 8.476; P = 0.005) were significantly correlated with survival, which was an independent risk factors for prognosis. Tumour maximum diameter (HR 1.608; 95% CI 0.770–3.360; P = 0.206) and MGMT methylation (HR 2.597; 95% CI 0.799–8.441; P = 0.112), on the other hand, had no effect on survival in GBM patients.

## Discussion

More than 80% of primary malignant brain tumors are gliomas, the most prevalent primary central nervous system tumors in adults^3^. Gliomas are categorized into grades 1–4 in the 2021 WHO Classification of Tumours of the Central Nervous System, with grades 1 and 2 being low-grade gliomas and grades 3 and 4 being high-grade gliomas^16^. According to the WHO classification, grade 4 gliomas, or glioblastomas, have an incidence rate of roughly 3.1 per 100,000 and account for 45.6% of initial malignant brain tumors. GBM is the most malignant and very vulnerable to recurrence, although having a low incidence rate. Patients often have a terrible prognosis, with a median survival of less than 2 years^3^.

A number of widely used prognostic factors for gliomas, including tumor grade, pathological classification, and degree of differentiation, are challenging to obtain prior to surgery, and other easily accessible factors, like age, gender, number of tumors, and blood-related parameters, are not very effective in predicting a patient's prognosis with glioma. Specifically, the evaluation of a patient's physical state before treatment is frequently predicated on the subjective judgment of the physician, which not only increases inter-observer variability but also lowers the precision of survival prediction. Thus, the need for more conclusive, trustworthy, impartial, quantifiable, and readily available prognostic markers is critical.

Primary sarcopenia usually refers to loss of skeletal muscle mass associated with aging, whereas secondary sarcopenia is associated with a variety of adverse factors, including disease. Research has demonstrated that various aspects of the frailty phenotype, such as skeletal muscle mass, functional status, medical comorbidities, and nutritional status, are ultimately linked to secondary sarcopenia^17^. A physiological state of diminished reserve, frailty is known to be a strong predictor of poor postoperative results in surgical patients^18^, especially those with brain tumors^19^.

It is also linked to an increased risk of unfavorable clinical outcomes. Consequently, the prognosis of patients is frequently determined in the clinic using sarcopenia as a component of frailty.Oncology research on the correlation between sarcopenia and cancer prognosis is expanding. In cancer patients, sarcopenia can co-occur with cachexia, which is characterized by severe weight loss and loss of skeletal muscle and adipose tissue^20^. Although considered two separate diseases, the pathophysiology of sarcopenia and cachexia are overlapping, both are multifactorial and include a misbalance between lower protein synthesis and higher protein degrada tion because of an elevated intracellular inflammation and oxidative stress^21^. The pathophysiological mechanism of the association between sarcopenia and poorer outcomes in cancer patients has remained relatively uncertain. Recent findings suggest five possible mechanisms: the role of skeletal muscle mass to modulate inflammation through the immune system via cytokines and myokines, the influence on insulin-dependent glucose control, mitochondrial function, synthetic and degradative protein pathways, and pharmacokinetics of anticancer drugs^22^. In metastatic and non-metastatic breast cancer, the occurrence of sarcopenia is associated with an increased risk of death and reduced OS in patients^23,24^. The correlation between sarcopenia and survival has also been demonstrated in hepatocellular, pancreatic, ovarian, gastric, and oesophageal cancers^25–29^.

Malignancy can result in a hypercatabolic state brought on by systemic inflammation and other tumor-related variables, which accelerate the progression of skeletal muscle wasting and sarcopenia^30^. The presence of sarcopenia indicates that patients have limited reserves to deal with surgical stress and are more prone to complications^31^, and higher mortality^32^. Several studies have shown that L3-SMI correlates well with patients' whole-body skeletal muscle mass and has become an objective and valid evaluation index of patients' whole-body muscle mass^33^. In clinical practice, it is more common to use the third lumbar skeletal muscle index (L3-SMI) to determine muscle mass. The L3-SMI is calculated by obtaining a cross-section at the L3 level by computed tomography (CT) and then measuring the skeletal muscle area (SMA) by the threshold method and dividing by the square of height^34^. Recent research has demonstrated that cephalocervical skeletal muscles such as TMT can also be used to detect muscle mass decrease in addition to lumbar skeletal muscles^35^. Moreover, according to the consensus of the American Society for Parenteral and Enteral Nutrition, temporal muscle atrophy is one of the physical manifestations of muscle atrophy in malnutrition and can be used in the nutritional assessment of patients which further substantiates the viability of employing TMT as an assessment tool for evaluating total body muscle mass^36^.

Temporal muscle thickness has been shown to be a suitable surrogate marker of skeletal muscle mass and can be used as a potential basis for determining sarcopenia^10,11^. Studies looking at the relationship between survival time and thickness of the temporalis muscle in patients with brain metastases have been conducted recently, and the results indicate that the thickness of the temporalis muscle can be used as an independent parameter to predict survival and frailty in patients with brain tumours^12,13^. Thus, in the era of molecular typing of gliomas, this study explores the predictive relationship between TMT and IDH wild-type glioblastoma patients and focuses on the prognostic relevance of TMT in glioblastoma patients based on prior research.

In recent years, there have been the following observations in studies exploring the potential role of TMT as a surrogate marker for sarcopenia in the prediction of survival in patients with GBM.Hsieh et al. analysed TMT measured by postoperative CT in 87 patients with newly diagnosed GBM, and found that TMT correlated with OS in patients with GBM, and that those with a thicker TMT had a greater median OS than those with a thinner TMT^37^. A study by Huq et al. found that TMT was associated with important prognostic variables in GBM and predicted postoperative survival in patients with progressive GBM^38^. Furtner et al. analysed cranial MR images of 596 patients with GBM and showed that patients with TMT higher than the cut-off performed better in terms of OS and PFS, and pointed out that TMT was an independent prognostic factor in patients with progressive GBM^14^, moreover, their recent study again pointed out that TMT can also be used as a reference indicator for the prognosis of newly diagnosed GBM patients, and TMT thinning suggests that patients have a poor prognosis^15^. Liu et al. retrospectively analysed the database of 130 primary GBM patients, and found that the survival time of the patients with TMT higher than the median group was significantly longer than that of the patients with TMT lower than the median group, and the results showed that the TMT, as an independent predictor of survival prediction of primary GBM patients, is sensitive and has the ability to predict the survival of patients with primary GBM. The results showed that TMT as an independent predictor of survival in patients with primary GBM is sensitive and has the potential to predict the survival time of patients^39^. A Meta-analysis by Sadhwani et al. also came to the same conclusion as the former^40^. We reached the same conclusion in our present study, further providing clinical validation of the theory. The results of this study showed that the median survival time of 102 patients was 14 months (range: 2–85 months), and the median overall survival of patients in the TMT > median group (23.0 months) was significantly increased compared to the median overall survival of patients in the TMT ≤ median group (9 months) (P < 0.001; Log-rank test). The results of multifactorial regression showed that temporalis muscle thickness was an independent prognostic factor in patients with glioblastoma. The novelty of this study is that in the era of molecular staging of glioblastoma, TMT also serves as an independent predictor of IDH wild-type glioblastoma. Survival analysis in 45 patients with IDH wild-type glioblastoma showed a significant increase in median overall survival (12 months) in patients in the TMT > median group compared with median overall survival (8 months) in patients in the TMT ≤ median group, (P < 0.001; Log-rank test), and multifactorial regression results showed that temporalis muscle thickness was an independent prognostic factor.

However, some scholars have come to the opposite conclusion. a multicentre analysis by Wende et al. showed that TMT can only be used as a surrogate parameter for other epidemiological data in patients with newly diagnosed GBM, and that their data do not yet support the role of TMT as an independent prognostic marker^41^. Klinigenschmid et al. study found that TMT has no prognostic relevance in patients with high-grade glioma compared to functional scales^42^. Similar conclusions were reached by Muglia et al., who found that preoperatively measured TMT in patients with newly diagnosed and untreated GBM did not significantly correlate with patient prognosis, age at the time of surgery, or preoperative performance status, and that its validity for prognostic judgement may be limited to patients with brain metastases and recurrent or treated GBM^43^. Of course both the Wende et al. and Muglia et al. studies were single-centre retrospective studies with sample sizes of less than 100, which increases selection bias and information bias in the subjects enrolled in the experiment.

The incidence of GBM increases with age, from 0.15/100,000 in childhood to a peak of 15.03/100,000 at 75–84 years of age. Its survival rate is inversely proportional to age, with an average 5-year survival rate of < 5% in patients with GBM, including a 5-year survival rate of < 2% in patients over 65 years of age^3^. In this study, we discovered that in a univariate analysis of age and overall survival in 102 patients (HR 1.029; 95% CI 1.013–1.046; P < 0.001), age was related to the prognosis of glioblastoma. However, in a multivariate analysis of the age at diagnosis (HR 1.013; 95% CI 0.996–1.031; P = 0.142), this conclusion was not reached. This could be attributed to the sample size and the fact that the multifactorial analysis had more confounders. Studies have also revealed a gender association between GBM occurrence and the fact that men are more likely than women to develop GBM.Furthermore, research has indicated a correlation between the incidence of GBM and gender, with a greater incidence rate in men than in women^44^. In our study, we discovered that the incidence was higher in males than in females across the 102 patients, 56 (54.9%) of whom were males and 46 (45.1%) of whom were girls. Prognostically speaking, however, 102 patients showed no difference in prognosis based on sex (HR 0.956; 95% CI 0.642 to 1.422; P = 0.823).The Stupp regimen is now considered the standard first-line treatment option for glioblastoma, consisting of maximal surgical resection supplemented by postoperative radiotherapy combined with temozolomide (TMZ) chemotherapy^2^. Glioblastoma patients' prognosis was significantly improved with the introduction of standard treatment, and radiotherapy combined with concurrent temozolomide chemotherapy (HR 0.477; 95% CI 0.272 to 0.834; P = 0.009) was an independent risk factor that affected the prognosis of GBM patients and was significantly associated with survival in 102 patients. In 45 patients with IDH wild-type glioblastoma, postoperative radiation therapy combined with concurrent temozolomide chemotherapy was similarly substantially linked with survival (HR 0.110; 95% CI 0.029 to 0.422; P = 0.001).This cohort, consisting of 45 IDH wild-type glioblastoma patients, was very small but homogeneous, with 40 of them (88.9%) receiving concurrent and simultaneous postoperative radiation and temozolomide chemotherapy. We discovered that TP53 mutations in this cohort did not predict GBM (P = 0.059; Log-rank test). The incidence of TP53 mutations in GBM has been shown to range from 25 to 37%. This study confirms the finding that TP53 mutations indicate a worse prognosis for low-grade gliomas but have no meaningful predictive value for GBM^45^.

Potential confounding factors that may alter the survival relationship between TMT and GBM must be taken into account while examining the survival relationship and prognostic significance of TMT and GBM. The following are possible confounding variables that could influence the connection between TMT and GBM survival. The patients' first characteristics come first. Since the sex-specific TMT thresholds used in some studies are based on standardised reference values for healthy Caucasians, it is not possible to rule out the possibility that potential racial differences in muscle mass could have an impact on the prognosis of TMT and GBM. Furthermore, the majority of current clinical retrospective studies do not include information on patient race^37^. To investigate prognostic disparities between GBM patients of different races, more thorough future research with patients of other races is required. Although there was formerly a consensus that TMT thins with age, subsequent studies have demonstrated no significant correlation between age and TMT, casting doubt on the association between patient age and GBM prognosis^37–39^. The distinction between real age and biological age helps to understand this. Biological age is thought to have a higher link with death than actual age because it more correctly represents the patient's level of debilitation and provides a more realistic assessment of the patient's current physical condition, as shown by numerous research^46^. In order to assess a patient's fragility, biological age rather than chronological age should be taken into consideration. The biological age of the patient can be employed as a stratification factor in future trials to evaluate the efficacy of treatments that target the patient's level of debilitation, based on the independent prognostic role of TMT. The patient's reaction to the treatment is the next consideration. Research indicates that individuals who have a low TMT (those who are at risk of sarcopenia) are more likely to experience an early pregnancy termination and are much less likely to receive second-line therapy in the event of progressive or recurrent GBM. These factors have a negative impact on the patient's prognosis^47^. Although there is no discernible relationship between TMT values and variations in the length and dosage of corticosteroid administration in the current investigation^15^, it has been proposed that corticosteroid treatment may accelerate the loss of muscle mass in patients with recurrent GBM^14^. Thus, to further investigate the relationship between TMT and steroid medication, more thorough prospective studies are required in the future.In addition, radiotherapy itself can have an impact on TMT, with some studies noting that patients with lower baseline TMT experience TMT attrition during radiotherapy and have a higher risk of death^15^. Therefore, independent studies are needed to further validate changes in TMT before and after patients receive radiotherapy. Finally, multiple comorbidities can also have an impact on the prognostic relationship between TMT and GBM. The most common is the effect of oral-related disorders. Studies have shown that patients have thinner muscles, including the temporalis muscle, due to lack of chewing compared to the general population^11,14^. Thus, the outcomes of research examining the association between TMT and GBM prognosis may be affected by the inclusion of individuals with the aforementioned diseases. Nowadays, the majority of clinical trials do not initially obtain a patient's history of oral disease. Instead, the researcher measures the patient's bilateral TMT and averages it for analysis, thereby excluding as much influence from oral disease as possible. We also measured and averaged the patients' bilateral TMT in the current investigation. Nonetheless, there is still value in examining the association between TMT and prognosis in GBM patients due to the influence of oral illness history on TMT.

TMT has been shown in recent years to be correlated with measures of frailty such grip strength, dietary status, and general functional status^10^. Thus, in the future, TMT could be considered, in along with myasthenia gravis in other surgical specialties, as part of a more comprehensive preoperative evaluation of frailty in neurosurgical oncology to determine a patient's prognosis. The therapeutic application of TMT has several benefits, especially in the field of neurosurgery. First of all, TMT is simple to measure because the majority of patients with brain tumors have regular MR images of their brains taken. This makes it easier for doctors to take quick measurements using MR images that have already been obtained in the clinic rather than the more difficult measurements of cross-sectional area at the L3 level.Second, TMT is more practically useful because it is unaffected by radiation-induced atrophy or muscle oedema; instead, it is influenced only by oral illness or prior surgery^11,14^. Furthermore, research has demonstrated that TMT measures exhibit greater consistency and less variability among various measurers, hence rendering it more suitable for incorporation into therapeutic practices^12,37^. TMT might be thought of as a sarcopenia measure that can be realistically included into current neurosurgical processes to direct the perioperative care of GBM patients and, ideally, forecast patient survival.

In terms of their physical state and surgical tolerance, clinicians can distinguish between patients based on their preoperative TMT; patients with TMT above a particular threshold can undergo surgery directly, while patients with TMT below this threshold should be advised to undergo a brief perioperative optimization. While it is important to avoid postponing GBM surgery too long, even a short period of perioperative optimization can partially offset the unsatisfactory results of immediate surgery. A multidisciplinary strategy is used for this kind of optimization, which includes medical co-morbidity care, physiotherapy, and nutritional supplementation^48–50^. Furthermore, a patient's decreased TMT indicates a higher chance of sarcopenia. Research has demonstrated that patients with GBM who are at risk for sarcopenia are more likely to discontinue Stupp therapy early and are less likely to receive second-line therapy when a recurrence occurs^47^. This means that doctors can modify therapy promptly in response to changes in a patient's TMT, resulting in a more suitable and personalised treatment plan for the patient. According to TMT, doctors may advise patients who require it to continue nutritional supplements and physical therapy during the postoperative phase of treatment in order to preserve function following radiation and chemotherapy. These measures may improve patients' quality of life and postoperative survival^14,51^, and thus deserve further research by scholars on TMT and its clinical application.

This study also has some limitations. Firstly, there is a lack of some important information related to molecular pathology data in patients from 2010 to 2016, which may have influenced the survival time of GBM, such as MGMT, TP53, etc. Then, lies in the fact that it is a single-centre retrospective study with a small sample size, and due to the limitations of retrospective study methodology, there may be selection bias and information bias, and any pathology-related information that has not been collected may affect the results of the study. Although all eligible glioma cases in our institution have been retrospectively included in recent years, there is a lack of external validation and larger, multicentre and prospective studies are needed to confirm these results. Secondly, any factor that may affect temporalis muscle thickness can influence the relationship between TMT and the prognosis of patients with primary glioblastoma. As in the talk we have analysed the baseline characteristics of the patients, the response of the patients to the treatment, the multiple comorbidities and other relevant factors on the potential impact of this study.

Notwithstanding the study's limitations, our analysis of the data and conclusions led us to the conclusion that temporal muscle thickness is a possible predictive factor for glioblastoma and a valuable surrogate marker of skeletal muscle volume and function. TMT measurement using MR images may be automated in the future by combining it with deep learning models, artificial intelligence, etc., and integrating it into the clinical workflow. This will decrease measurement heterogeneity, streamline clinical procedures, increase measurement efficiency and accuracy, and enable doctors to dynamically track changes in TMT during patient follow-up to assess prognosis.

## Materials and methods

### Clinical information

From 30 May 2010 to 30 June 2020, with the last follow-up on 10 February 2023, clinical and pathological data, temporal muscle thickness measured on magnetic resonance, and prognosis of 102 patients with a histological diagnosis of glioblastoma were collected in the Department of Oncology Radiotherapy of the First Affiliated Hospital of Dalian Medical University.The molecular typing data of glioblastoma IDH typing, MGMT methylation, and TP53 were present in the clinical data of 55 cases from 1 April 2016 to 30 June 2020. Of them, 45 patients had IDH wild-type glioblastoma, and these 45 patients underwent separate analysis.

Inclusion standards: (1) Each patient had a recent histology diagnosis of glioblastoma. (2) Pathological confirmation by craniotomy or stereotactic biopsy (3) Complete and accessible preoperative brain MR imaging data as well as follow-up data (4) Patient age > 18 years (5) Surgical resection without temporal region involvement. Age 18 years, history of radiotherapy, chemotherapy, and/or brain surgery, and absence of a known cause of death or last follow-up report are all considered exclusion factors.

### The thickness of the temporal muscle

**Figure 3:**
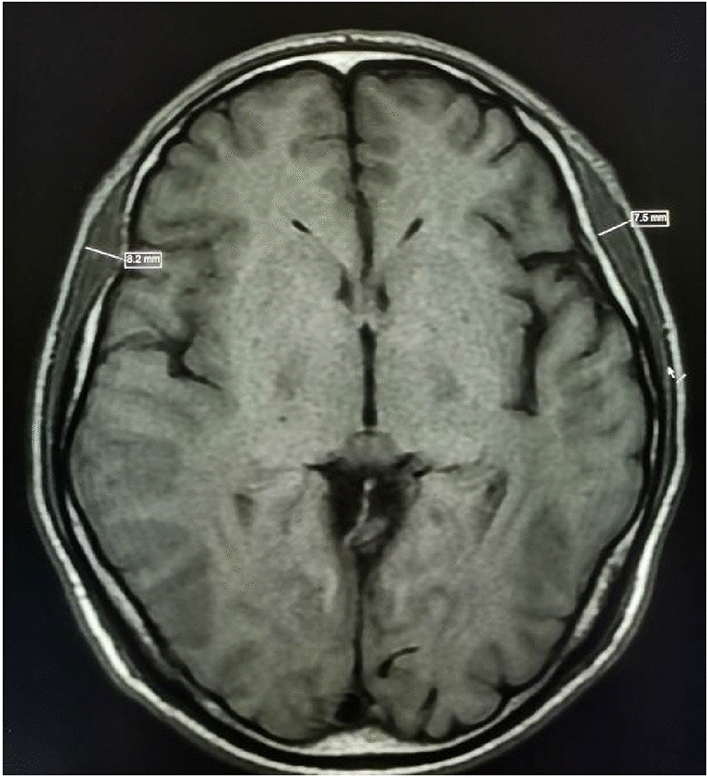
A 52-year-old male patient with an overall survival of 47 months (bilateral mean TMT = 7.85 mm).

**Figure 4:**
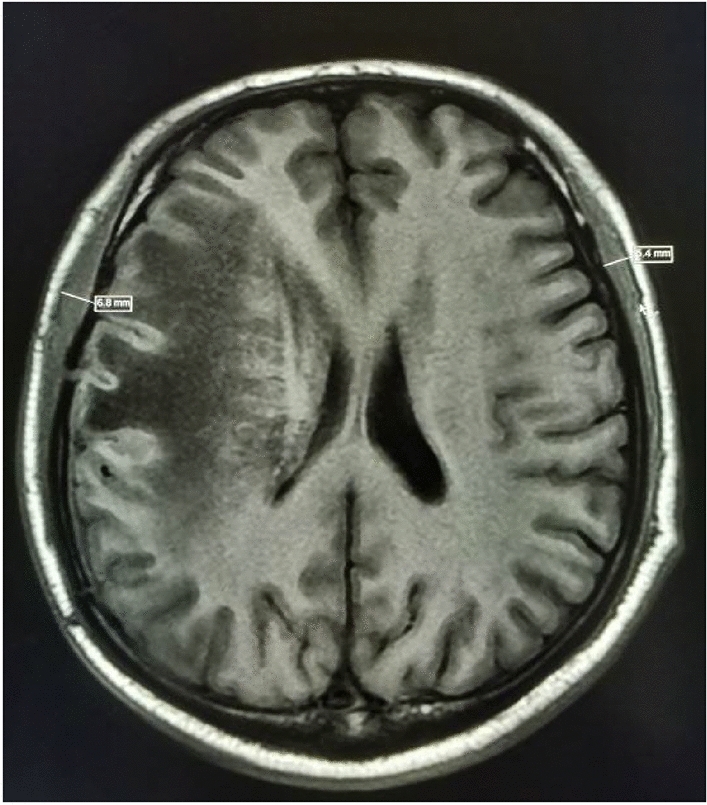
A 69-year-old female patient with an overall survival of 7 months (bilateral mean TMT = 6.10 mm).

Imedpacs image Viewer software (Version 4) was used to evaluate TMT on preoperative T1-weighted axial contrast-enhanced brain MR images. The supraorbital fissure and the orbital apex can be used as anatomical landmarks during TMT measurements, according to earlier research^14^. All patients had their right and left temporalis muscles measured perpendicular to the long axis at the level of the orbital apex. Each patient's left and right sides' TMT were added together, and the resulting number was multiplied by two to determine the patient's assessed TMT^14^. The rest of the patient's clinical information was kept a secret from the radiologist, who did all measures.The images obtained from the temporalis muscle thickness measurements on MR are shown in Figs. 3 and 4.

### Data evaluation

Statistical analysis and data visualization were performed using SPSS version 27.0 (IBM Corporation, Armonk, NY, USA).Patients were split into two groups based on median and TMT thresholds were established using median analysis. Chi-square tests were used to evaluate differences in categorical variables, and descriptive and frequency statistics were generated to summarize demographic and clinical data. The evaluation of independent prognostic markers was done using univariate and multivariate Cox regression models. The Kaplan–Meier method was then used for survival analysis, and the log-rank approach for statistical significance analysis. Statistics were deemed significant at *p* 0.05.

### Regulatory approval

All participants provided written informed consent before the examination. The present study was reviewed and approved by the Ethics Committee of the First Affiliated Hospital of Dalian Medical University (2023–225). All methods were carried out in accordance with the principles expressed in the Declaration of Helsinki.

### Supplementary Information

Supplementary Information.

## Data Availability

All data generated or analysed during this study are included in this published article and its Supplementary Information files.
